# Wernicke encephalopathy: a mini review of the clinical spectrum, atypical manifestations, and diagnostic challenges

**DOI:** 10.3389/fneur.2025.1566366

**Published:** 2025-07-16

**Authors:** Siping Li, Chengzhi Xing

**Affiliations:** ^1^Department of Ultrasound Medicine, Pu’er People’s Hospital, Pu’er, China; ^2^Department of Neurology, Pu’er People’s Hospital, Pu’er, China

**Keywords:** Wernicke encephalopathy, thiamine deficiency, atypical manifestations, diagnostic challenges, neuroimaging

## Abstract

**Background:**

Wernicke encephalopathy (WE) is a severe neurological disorder caused by thiamine (vitamin B1) deficiency. Though often associated with chronic alcohol abuse, it can also arise from other conditions that impair thiamine intake or absorption. The classic triad of symptoms includes ophthalmoplegia, an abnormal mental state, and gait ataxia, although these may not be present in all patients, leading to underdiagnosis and undertreatment.

**Methods:**

This mini review synthesizes data from clinical studies, autopsy reports, and imaging findings to assess the prevalence, diagnostic challenges, and treatment protocols for WE. It examines the role of various etiological factors, the presentation of atypical symptoms, and the utility of diagnostic tools such as magnetic resonance imaging (MRI) and vitamin B1 assays.

**Results:**

Autopsy studies report a prevalence of WE that ranges from 0.4 to 2.8%, with most patients experiencing alcohol addiction or having other alcohol use disorders. The diagnosis of WE is primarily clinical, based on the Caine criteria in which a person must display at least two of the three classic symptoms or evidence of nutritional deficiency. MRI is a valuable tool for diagnosing WE, typically showing symmetric T2/FLAIR hyperintense signals in specific brain regions. Treatment involves prompt thiamine replacement, with intravenous administration being the most effective method to ensure adequate brain uptake.

**Conclusion:**

WE remains a challenging condition to diagnose due to its variable presentations and the potential for atypical symptoms. Early recognition and treatment with thiamine are crucial to prevent irreversible neurological damage or death. There is a need for clear diagnostic and treatment guidelines to improve the management of WE and reduce the risk of underdiagnosis and undertreatment.

## 1 Introduction

Wernicke encephalopathy (WE), a metabolic brain disorder precipitated by thiamine (vitamin B1) deficiency, presents a formidable diagnostic and therapeutic challenge in contemporary medicine (1). Predominantly associated with chronic alcohol abuse in developed nations, WE also emerges from a spectrum of conditions that compromise thiamine intake or absorption, such as malnutrition and gastrointestinal surgeries (2). The latter, in particular, heightens the risk of thiamine malabsorption, underscoring the need to consider surgical histories as potential etiological factors in WE’s pathogenesis (3, 4).

Despite the well-established link between chronic alcoholism and WE, a significant proportion of patients diagnosed with the condition lack a history of alcohol misuse, highlighting the complexity of its etiology (5, 6). International research underscores the alarmingly low pre-mortem diagnosis rate of WE, with only 20% of cases identified antemortem, often discovered post-mortem during autopsy (7, 8). This figure is even more disconcerting for non-alcoholic WE, where the misdiagnosis rate is alarmingly high, with a pre-mortem diagnosis rate of merely 16% (8, 9). The classic clinical triad of WE—encephalopathy, ophthalmoplegia, and ataxia—serves as a diagnostic hallmark. However, the frequency of each component in the triad varies. Encephalopathy is the most common, present in approximately 82% of patients, as it results from the involvement of thalamus nuclei, mammillary bodies, and the brainstem reticular activating system, which are crucial for maintaining normal mental states (10). Ophthalmoplegia, such as horizontal or vertical nystagmus, eye muscle paralysis, and diplopia, occurs in around 29% of cases (11). This is due to the damage of cranial nerve nuclei in the brainstem, like the oculomotor nerve nucleus, abducens nerve nucleus, and vestibular nerve nucleus, which control eye movements. Ataxia is seen in about 23% of patients and is caused by the dysfunction of the cerebellar vermis and vestibular function, which are essential for maintaining balance and coordinating movements. The full triad is observed in a mere 16% of patients, with the majority exhibiting only one or two components (12). It is significant that 19% of patients present without any of the classic symptoms, often manifesting with rare and atypical symptoms, particularly in the early stages of non-alcoholic WE. This underscores a critical gap in clinical recognition of WE’s diverse presentations, where reliance on the classic triad can lead to missed or delayed diagnoses (13, 14).

The consequences of diagnostic oversight are profound, with delayed diagnosis and treatment potentially leading to irreversible neurological damage or even fatal outcomes. This mini review aims to bridge this knowledge gap by exploring the full spectrum of clinical evolution and atypical manifestations of WE. By scrutinizing the nuances of its presentation, this work endeavors to enhance clinical acumen, thereby improving diagnostic accuracy and patient outcomes in the management of this under-recognized but devastating condition.

## 2 Epidemiology and etiology

Autopsy studies have reported the prevalence of Wernicke encephalopathy (WE) as ranging from 2 to 3%, yet the clinical diagnosis rate is significantly lower, at 0.06 to 0.13%. The age of onset varies, with infants typically affected between 2 to 5 months and adults between 30 to 70 years, with an average age of 42.9 years and a slight male preponderance (6). The gender distribution among WE patients is approximately 1.7:1 in favor of men (8). Regional variations in prevalence are noted, with rates of 1.7 to 2.8% in Australia, 0.5% in the UK, and 0.4 to 1.4% in France (15).

The etiology of WE is multifactorial, with chronic alcoholism being a leading cause. Other causes include persistent vomiting during pregnancy, malnutrition following gastrointestinal surgery, and severe malabsorptive conditions such as malignancies, Crohn’s disease, and major burns. The underlying mechanism in all these scenarios is a sustained deficiency in thiamine intake or absorption, which is the root cause of WE. Thiamine, or vitamin B1, is essential and cannot be synthesized *de novo* in the human body; it is primarily derived from dietary sources, with body stores sufficient for approximately 18 days (11).

In patients with alcoholism, several pathomechanisms make these patients susceptible to Wernicke encephalopathy. Firstly, chronic alcohol consumption often leads to poor dietary habits. People with alcohol dependency tend to have a diet that is low in essential nutrients, including thiamine, which directly reduces thiamine intake (16). Secondly, alcohol can disrupt the normal absorption of thiamine in the gastrointestinal tract. It may damage the intestinal mucosa, interfering with the active transport of thiamine across the enterocytes, thus impairing thiamine absorption (17). Thirdly, alcohol metabolism in the liver can deplete the body’s thiamine stores. The process of metabolizing alcohol requires thiamine-dependent enzymes, and excessive alcohol intake leads to an increased demand for thiamine, which cannot be met due to insufficient dietary intake and impaired absorption, eventually resulting in thiamine deficiency (18).

Thiamine deficiency, the primary cause of WE, is most commonly associated with chronic alcohol abuse. However, non-alcoholic causes of WE are increasingly recognized (17, 19). The most frequent non-alcoholic etiologies include hyperemesis gravidarum and post-gastrointestinal surgery (20). Other common causes encompass conditions leading to frequent vomiting, chronic diarrhea, cancer, chemotherapy, renal diseases, febrile illnesses, chronic infections, HIV/AIDS, and hyperthyroidism, among other systemic diseases (21).

Thiamine is a precursor to thiamine pyrophosphate (TPP), a crucial coenzyme in the tricarboxylic acid cycle. Its deficiency can lead to impaired glucose metabolism, resulting in brain tissue lactic acidosis, endothelial cell dysfunction, and disruption of neurotransmitter synthesis, release, and uptake. These metabolic derangements induce neuronal edema and apoptosis, culminating in selective damage to metabolically active neurons in regions such as the periventricular and periaqueductal gray matter, manifesting as clinical symptoms (22, 23).

## 3 Atypical clinical manifestations

### 3.1 General overview

The classic clinical presentation of Wernicke encephalopathy (WE), resulting from thiamine (vitamin B1) deficiency, includes the triad of acute mental status changes, ocular motor abnormalities, and ataxia. However, the full triad is observed in only 10.0 to 16.5% of patients, with the majority exhibiting a variety of mental state alterations, including confusion, disorientation, dizziness, somnolence, apathy, cognitive dysfunction, memory and attention deficits, and in severe cases, coma and death (24). Studies indicate that the clinical triad presents differently in alcoholic versus non-alcoholic WE, with a minority of patients in both groups exhibiting the full triad (25). This section delves into the atypical clinical symptoms of WE, highlighting the complexity and heterogeneity of its manifestations.

Literature reports of rare clinical manifestations of WE include the following: (1) rare neurological manifestations: vision loss, optic disc edema, retinal hemorrhage, unequal pupils, sluggish light reflex, light-near dissociation, hearing loss, epileptic seizures, flaccid paralysis of the limbs or lower limbs, dysarthria, dysphagia, chorea, tremor, and sensory abnormalities; (2) non-neurological manifestations: hypothermia, fever, hypotension, tachycardia, bradycardia, gastrointestinal reactions, and refractory hyponatremia. In the absence of large-sample prospective studies, it is not possible to determine the exact frequency of these clinical manifestations, and only a few cases have been reported (Table 1).

**Table 1:** Rare clinical manifestations of WE.

Rare clinical manifestations of WE	Possible affected areas or causes
Rare neurological manifestations	
Vision loss	Optic disc edema
	Retinal hemorrhage
	Damage to the afferent visual pathway (thalamus, cortex)
Unequal pupils, sluggish light reflex, light-near dissociation	Rostral tegmental area
Hearing loss	Damage to the afferent auditory pathway (thalamus, inferior colliculus)
Seizures	Cortical metabolic abnormalities, glutamate-mediated excitotoxicity, oxidative stress
Flaccid paralysis of limbs or lower limbs, dysarthria, dysphagia	Acute polyneuropathy
Movement disorders (chorea, tremor)	Subthalamic nucleus
Sensory abnormalities (hypoesthesia, pain)	Acute polyneuropathy
Non-neurological specific manifestations	
Hypothermia	Hypothalamus
Fever	Hypothalamus
Hypotension, tachycardia, bradycardia	Hypothalamus
Gastrointestinal symptoms (anorexia, nausea, vomiting)	Hypothalamus
Refractory hyponatremia	Hypothalamus

### 3.2 Ocular manifestations

Visual impairment in WE is a consequence of the involvement of the afferent visual pathway and can range from sudden blindness to gradual visual deterioration over days (typically bilateral and without ocular pain). Two mechanisms have been proposed for vision loss in WE: (1) funduscopic abnormalities such as optic disc edema, retinal hemorrhages, and peripapillary nerve fiber layer thickening, and (2) central visual pathway involvement with lesions in the optic chiasm, lateral geniculate body, or occipital cortex, as indicated by magnetic resonance imaging (MRI) findings (11, 26, 27).

After treatment with vitamin B1, most patients experience an improvement in ocular manifestations. In many cases, visual acuity starts to improve within a few hours to days. However, in some patients, especially those with more severe or long-standing damage, residual visual deficits may remain. The frequency of patients with residual ocular symptoms is difficult to precisely determine, but it has been estimated that a significant proportion, around 20–30% based on some case series (16), may have persistent mild visual impairments such as reduced contrast sensitivity or subtle visual field defects.

### 3.3 Auditory impairment

Auditory disturbances in WE are less common, with a higher incidence in non-alcoholic WE, particularly among young women (28). The mechanism involves central auditory pathway impairment, with symmetrical involvement of the inferior colliculi and thalami affecting signal transmission, leading to sensorineural hearing loss (29, 30). Brainstem auditory evoked potential changes are sensitive indicators of thiamine deficiency and can aid in early diagnosis and treatment monitoring (31).

Upon treatment with vitamin B1, the prognosis for auditory impairment in WE patients is relatively favorable. Approximately 40% of patients experience a resolution of hearing loss within 2 weeks. However, about 50% of patients may still have some degree of residual hearing loss, which may persist for up to 3 months or longer in some cases (32). These patients may require long-term follow-up and possible audiological rehabilitation.

### 3.4 Seizures

Seizures in WE are rare, occurring in 0–3.1% of cases, and are more commonly seen in non-alcoholic WE, where they can be an initial symptom (33). The seizures are typically generalized tonic-clonic, but partial seizures and status epilepticus can also occur (34). The pathogenesis is likely related to cortical metabolic abnormalities and excitotoxicity due to glutamate excess and oxidative stress (35).

Following vitamin B1 treatment, seizures in WE patients rarely recur. In most cases, once the thiamine deficiency is corrected, the risk of further seizures significantly decreases. However, in a small number of patients, especially those with pre-existing brain damage or other underlying neurological conditions, there may still be a slightly increased risk of future seizure activity, although the exact frequency is not well-defined in the literature (36).

### 3.5 Peripheral neuropathy

Acute polyneuropathy associated with WE is infrequently reported, particularly in non-alcoholic cases. It presents as a progressive, symmetrical, predominantly distal sensorimotor polyneuropathy, with areflexia and sensory disturbances (37, 38). The pathophysiology may involve reduced activity of pyruvate dehydrogenase due to thiamine deficiency, leading to decreased ATP synthesis and impaired Na^+^/K^+^-ATPase activity, essential for nerve depolarization and axonal excitability (39).

The course of recovery for peripheral neuropathy in WE patients after treatment is relatively slow. It may take several months to a year or more for significant improvement in muscle strength and sensory function. Many patients may experience some degree of residual neuropathy, with an estimated frequency of around 50–60% having persistent mild to moderate symptoms such as distal weakness or altered sensation (40). These patients may need continuous physical therapy and supportive care to manage their symptoms.

### 3.6 Movement disorders

Movement disorders in WE, including chorea and tremor, may result from involvement of subcortical structures such as the basal ganglia (37, 41). Hypothalamic involvement can present as hypothalamic syndrome, with early manifestations of hypothermia, which is associated with a higher mortality risk in WE (42). Other features include hypotension, tachycardia, bradycardia, anorexia, nausea, vomiting, and hyponatremia, with hyperthermia possible in later stages (43, 44).

After treatment with vitamin B1, movement disorders in WE patients often show improvement. Chorea and tremor symptoms may start to subside within a few days to weeks. However, like other manifestations, some patients may have residual movement abnormalities. The frequency of patients with residual movement disorders is not precisely known but may be around 30–40% based on limited case reports (27, 37). For patients with hypothalamic-related symptoms, the prognosis depends on the severity of the initial insult. Hypothermia usually resolves relatively quickly with treatment, but other symptoms such as hypotension or electrolyte imbalances may require longer-term management, and a small proportion of patients may have persistent hypothalamic-related complications.

## 4 Diagnostic investigations

The diagnosis of Wernicke encephalopathy (WE) primarily follows the criteria established by the European Federation of Neurological Societies (EFNS) in 2010: (1) dietary deficiency; (2) ocular signs; (3) cerebellar dysfunction; (4) mental status abnormalities or memory impairment. Suspicion of WE is heightened when at least two of these four criteria are met. A diagnosis of WE can be made in cases presenting with the classic clinical triad and a clear etiology of thiamine deficiency. However, as previously mentioned, the classic triad is often absent in clinical practice, and imaging studies, particularly MRI, along with laboratory tests for vitamin B1, provide crucial clues for the definitive diagnosis (45).

### 4.1 Computed tomography

Head computed tomography (CT) scans may reveal symmetric hypodensities around the mesencephalic aqueduct and the periaqueductal region of the thalamus in WE but are often negative during the acute phase (46).

### 4.2 Magnetic resonance imaging

Currently, MRI of the brain is considered the most valuable examination for diagnosing WE. It typically shows symmetric T1 hypointense and T2/FLAIR hyperintense signals in the medial thalamus, mammillary bodies, periaqueductal region, and cerebellum bilaterally (47, 48). A recent study by Hiraga et al. (49) on 12 consecutive patients with WE confirmed by low blood vitamin B1 levels showed that 50% of patients had abnormal signals in the mammillary bodies, 6/12 (50%) in the medial thalamus, and 7/12 (58.3%) in the dorsal midbrain. Additionally, 5/12 (41.7%) had abnormalities in the dorsal pons. Besides the typical sites, 6 out of 12 patients (50%) showed abnormalities at atypical sites such as the splenium of the corpus callosum (4 patients), fornix (3 patients), cerebral cortex (2 patients), cerebellar vermis (2 patients), and dorsal medulla (1 patient). This suggests that in cases of confirmed WE with low blood vitamin B1 levels, some areas like the corpus callosum, fornix, and cerebral cortex may be more frequently involved than previously thought. Compared to conventional T2WI sequences, FLAIR sequences are more sensitive and accurate for lesion detection and localization. In the acute phase, WE lesions are primarily characterized by cytotoxic edema, which impedes water molecule diffusion, and are hyperintense on diffusion-weighted imaging (DWI), aiding in the early diagnosis of WE. DWI is more sensitive than conventional T1WI and T2WI in detecting irreversible neuronal damage and distinguishing types of cytotoxic edema. The apparent diffusion coefficient (ADC) maps in the acute phase of WE lesions show low signals, possibly related to cytotoxic edema caused by neuronal and astrocytic energy metabolism damage and local lactic acidosis (48, 50). However, ADC maps may show low or slightly high signals, which could be due to the predominance of cytotoxic edema in the early stage (low ADC) and vasogenic edema in the late stage (high ADC) (51). With treatment, these lesions can decrease or disappear. The sensitivity of MRI for diagnosing WE is 53%, and the specificity is 93% (52); a normal MRI thus does not rule out WE.

It is important to note that the MRI features and lesion locations in WE are not pathognomonic, necessitating differentiation from other acute brain diseases with similar MRI changes. Especially when there is no evident thiamine deficiency or atypical neurological symptoms, WE must be distinguished from paraneoplastic syndromes, encephalitis, Miller Fisher syndrome, primary brain lymphoma, Leigh’s syndrome, Behçet’s disease, multiple sclerosis, paraneoplastic encephalitis, variant Creutzfeldt–Jakob disease, severe hypophosphatemia, and other metabolic encephalopathies (3). Reports have indicated that atypical MRI lesions in non-alcoholic WE patients require differentiation from metronidazole-induced encephalopathy (16).

### 4.3 Vitamin B1 assays

The most reliable method is the measurement of transketolase activity in whole blood or red blood cells (45). As described, thiamine is a precursor to TPP, and the TPP effect is determined by the percentage increase in transketolase activity in red blood cells with added TPP versus without. A higher TPP effect indicates a more severe thiamine deficiency. Generally, a TPP effect of ≥16% is considered insufficient, and ≥25% indicates deficiency. This method has been widely researched and improved in domestic laboratories for practical operation (49). Studies have shown that determining thiamine levels in serum using reversed-phase ultra-high-performance liquid chromatography-tandem mass spectrometry is feasible, enhancing the method’s utility and reliability for clinical and research applications (53).

### 4.4 Other auxiliary tests

If peripheral neuropathy is involved, electromyography may show corresponding changes. In patients with WE presenting with psychiatric or consciousness disturbances, electroencephalogram (EEG) often reveals mild-to-moderate abnormalities (3). Cerebrospinal fluid (CSF) examination is typically normal and can serve to differentiate from other encephalopathies. Untreated WE patients often have increased blood pyruvate and lactate levels (5), providing important diagnostic information. WE patients with a history of chronic alcoholism often have magnesium deficiency, and serum magnesium measurements can be of reference value.

## 5 Treatment and prognosis

WE is an emergency in neurology, and any delay in treatment can lead to permanent neurological damage, mandating prompt vitamin B1 therapy. There is currently only one insufficient randomized controlled trial providing a unified understanding of treatment dosage, administration route, and duration (17). The EFNS has issued recommendations for vitamin B1 treatment in WE (11): (1) apply in suspected or confirmed patients; (2) recommend intravenous vitamin B1 200 mg three times daily; (3) administer supplementation before carbohydrate use, followed by a normal diet; (4) continue treatment until clinical signs and symptoms no longer improve. Compared to non-alcoholic WE, alcoholic WE requires a larger dose of vitamin B1, and symptoms often do not fully recover. A vitamin B complex injection (Pabrinex), containing thiamine/vitamin B1 250 mg, vitamin B2 4 mg, vitamin B6 50 mg, and nicotinamide 160 mg, has been clinically applied in the UK. Guidelines (18) suggest that for suspected or confirmed WE, two vials of Pabrinex three times daily for 3 to 5 days may be administered. It should be noted, as recommended by the EFNS, that glucose should be prohibited before supplementing vitamin B1, as glucose metabolism continues to consume the deficient vitamin B1, exacerbating the condition. Magnesium, as a cofactor for thiamine-dependent enzymes and normal neurochemical transmission, when deficient, can reduce the effectiveness of thiamine; hence, magnesium supplementation is indicated. At this time, 35 to 50 mmol of magnesium sulfate and 1 L of 0.9% sodium chloride solution can be administered intravenously over 12 to 24 h (2).

If not treated promptly or treated improperly, the natural course of WE can progress, with patients presenting with coma, shock, and cardiovascular failure, indicating a poor prognosis. The mortality rate in untreated or improperly treated patients can reach up to 50% (17), and the majority of the remaining patients progress to Korsakoff’s syndrome, with irreversible neurological damage. Typically, ophthalmoplegia is the most recoverable, with ataxia and psychiatric disorders recovering more slowly, often improving within days to weeks (17), but most patients with WE with a history of alcoholism retain anterograde amnesia.

## 6 Conclusion

In summary, although the EFNS has issued diagnostic criteria for WE, the diversity and atypicality of its clinical manifestations, especially the increasing incidence of non-alcoholic WE in recent years, mean that a physician’s subjective experience plays a major role in clinical diagnosis and treatment, making missed and misdiagnoses highly likely. As a metabolic encephalopathy, particularly in its acute phase, WE can improve rapidly with early treatment. Therefore, this article aims to enhance the understanding of the disease among clinicians through the study of its clinical aspects, to avoid pitfalls in diagnosis and treatment, and to reduce the mortality and disability rates among patients with WE.
